# The evolution and age of populations of *Scaphinotus petersi* Roeschke on Arizona Sky Islands (Coleoptera, Carabidae, Cychrini)

**DOI:** 10.3897/zookeys.147.2024

**Published:** 2011-11-16

**Authors:** Karen Ober, Brian Matthews, Abigail Ferrieri, Sonia Kuhn

**Affiliations:** 1 Department of Biology, College of the Holy Cross, Worcester, MA 01610

**Keywords:** carabid ground beetles, divergence dates, phylogeography

## Abstract

Populations of the ground beetle *Scaphinotus petersi* are isolated in subalpine conifer forest habitats on mountain ranges or Sky Islands in southeastern Arizona. Previous work on this species has suggested these populations have been isolated since the last post-glacial maximum times as warming caused this cool adapted species to retreat to high elevations. To test this hypothesis, we inferred the phylogeny from mitochondrial DNA sequence data from several Arizona Sky Island populations of *Scaphinotus petersi* and estimated the divergence time of the currently isolated populations. We found two major clades of *Scaphinotus petersi*, an eastern clade and a western group. Our results indicated most mountain ranges form clades except the Huachucas, which are polyphyletic and the Santa Catalinas, which are paraphyletic. We estimated the Pinaleño population is much older than the last glacial maximum, but the Huachuca and Pinal populations may have been fragmented from the Santa Catalina population since the post-glacial maximum times.

## Introduction

Carabidae (ground beetle family) is one of the larger families of insects with approximately 40,000 described species (Lorenz 2005). The snail-eating beetles of the genus *Scaphinotus* belong to the carabid tribe Cychrini. Cychrines consist of about 150 species in four genera and are restricted to the Northern Hemisphere; the Cychrini genus *Scaphinotus*, found only in North America, began its initial radiation about 35 million years ago (Osawa et al. 2004, Scudder 1900) into 55 species (Lorenz, 2005). *Scaphinotus petersi* is a large ground beetle confined exclusively to moist coniferous forests that occur in southern Arizona at elevations > 1800 m. *Scaphinotus petersi* is a specialist predator of land snails, using elongated and narrow mouthparts to penetrate and extract the soft parts of terrestrial snails (Digweed 1993, LaRochelle 1972). *Scaphinotus petersi*, like other *Scaphinotus*, is flightless, with reduced or absent flight wings under fused elytra, and thus a poor disperser. Six subspecies of *Scaphinotus petersi* have been described (Ball 1966), and geographical variation among subspecies includes differences in size, head and neck characteristics, leg differences and color variation. All six *Scaphinotus petersi* subspecies live only on mountains in the sub-Mogollon area of Arizona, a region known as the Sky Islands.

The Sky Islands (Heald 1951), also called the Madrean Archipelago, are a unique complex of mountain ranges and ecosystems in southeastern Arizona. At present, hot, dry, desert grasslands and desert scrub in the valleys (the sea between the Sky Islands) act as barriers to the movement of upland forest species such as *Scaphinotus petersi* much as saltwater seas isolate flora and fauna on oceanic islands. As with oceanic islands, this separation of habitat limits genetic interchange between populations and creates environments with high evolutionary potential. The resulting Sky Island ecosystems, renowned for their biodiversity (Lomolino et al. 1989), support a high number of endemic species, including many threatened and endangered species, and are considered a biodiversity hot spot (Spector 2002). The Sky Islands are a natural laboratory in which to examine genetic differentiation and the evolutionary dynamics of vicariance. Mesic refuges, such as those in southwest mountains, may have been important centers of diversification during periods of dry climate for carabid beetles (Noonan 1992). Today, several Sky Island mountain ranges each contain a unique subspecies of *Scaphinotus petersi*.

The goal of this study was to infer the biogeographic history of *Scaphinotus petersi* in southeastern Arizona and investigate how the paleoclimatic oscillations of Quaternary affected the distribution of populations in the Sky Islands. We present a preliminary genealogy of mitochondrial DNA (mtDNA) sequences and use these data to address questions about population structure of this species and examine the potential role of the Pleistocene climate changes in the differentiation some of the Sky Island populations of *Scaphinotus petersi*.

## Methods

### DNA sequence data

**Figure 1.**
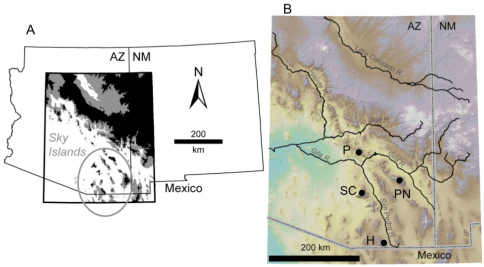
Study location **A** *Scaphinotus petersi* distribution is circled area. Habitat above 1830m is shown in black and between 1500 and 1830m is shown in grey **B** Shaded relief map of study area. Black dots denote sampling localities of *Scaphinotus petersi* used in this study (see Table 1) abbreviated as follows: P, Pinal Mountains; SC, Santa Catalina Mountains; PN, Pinaleño Mountains; and H, Huachuca Mountains. Figure courtesy of Sara Mitchell.

We collected DNA sequence data from 45 specimens of four of the six subspecies of *Scaphinotus petersi* in five localities in four mountain ranges (Table 1, Fig. 1). We included three outgroup species from the tribe Cychrini. One species of a related genus *Sphaeroderus*, and two other distantly related *Scaphinotus* species. Outgroup choices were limited by material available for DNA analysis. Genomic DNA was extracted following the protocol outlined in Maddison et al. 1999. PCR reactions were performed using a modification of the procedure described in Maddison et al. 1999. Reactions used a 53–56°C annealing temperature. This procedure was used to amplify approximately 1200bp of ND1 and adjacent RNA genes, and either a 500 bp portion or 1400 bps of COI. Macrogen Inc. (Korea) carried out DNA sequencing using an Applied Biosystems ABI 3730 48-capillary DNA analyzer with Big Dye Terminator Technology according to the manufacturer’s protocols (Applied Biosystems). The primers used for PCR amplification and DNA sequencing is given in Table 2. DNA sequence data was visualized using the SEQUENCHER 3.0 software (Gene Codes Corp.). Sequences were easily aligned by eye using MACCLADE 4.06 (Maddison and Maddison 2005). Data matrices are available from the corresponding author. Voucher specimens are in KAO insect collection at the College of the Holy Cross, Worcester, MA.

**Table 1.** Specimens, collection localities, and GenBank numbers included in this study.

**Specimen**	**Collection locality**	**Specimen number**	**COI GenBank**	**ND1 GenBank**
*Sphaeroderus lecontei*	MA: Worcester Co. Wachusett Reservior / 71.6849°W, 42.4048°N / 120m elev.	001	JN639333	JN641890
*Scaphinotus crenatus*	CA: Kern Co., Silvia Rd. / 37°29.789'N, 119°53.369'W	002	JN639334	JN641891
*Scaphinotus* sp.	CA: Kern Co. Hwy 49A / 37°22.806'N, 119°43.879'W	030	JN639335	JN641892
*Scaphinotus petersi grahami*	AZ:Graham Co., Pinaleño Mts., Columbine Corral Camp/Ash Creek / 32.7065°N, 109.9131°W elev. 2904m	040	JN639336	JN641893
*Scaphinotus petersi grahami*	AZ:Graham Co., Pinaleño Mts., Ladybug Trail / 32.6589°N, 109.8540°W elev. 2716m	041	JN639337	JN641894
*Scaphinotus petersi grahami*	AZ:Graham Co., Pinaleño Mts., Columbine Corral Camp/Ash Creek / 32.7065°N, 109.9131°W elev. 2904m	075	JN639369	JN641926
*Scaphinotus petersi grahami*	AZ:Graham Co., Pinaleño Mts., Columbine Corral Camp/Ash Creek / 32.7065°N, 109.9131°W elev. 2904m	076	JN639370	JN641927
*Scaphinotus petersi grahami*	AZ:Graham Co., Pinaleño Mts., Columbine Corral Camp/Ash Creek / 32.7065°N, 109.9131°W elev. 2904m	077	JN639371	JN641928
*Scaphinotus petersi grahami*	AZ:Graham Co., Pinaleño Mts., Columbine Corral Camp/Ash Creek / 32.7065°N, 109.9131°W elev. 2904m	078	JN639372	JN641929
*Scaphinotus petersi grahami*	AZ:Graham Co., Pinaleño Mts., Columbine Corral Camp/Ash Creek / 32.7065°N, 109.9131°W elev. 2904m	079	JN639373	JN641930
*Scaphinotus petersi biedermani*	AZ: Cochise Co., Huachuca Mts., Carr Canyon Trail / 31.4272°N, 110.3069°W elev. 2186m	044	JN639340	JN641897
*Scaphinotus petersi biedermani*	AZ: Cochise Co., Huachuca Mts., Carr Canyon Trail / 31.4272°N, 110.3069°W elev. 2186m	045	JN639341	JN641898
*Scaphinotus petersi biedermani*	AZ: Cochise Co., Huachuca Mts., Carr Canyon Trail / 31.4272°N, 110.3069°W elev. 2186m	046	JN639342	JN641899
*Scaphinotus petersi biedermani*	AZ: Cochise Co., Huachuca Mts., Carr Canyon Trail / 31.4272°N, 110.3069°W elev. 2186m	047	JN639343	JN641900
*Scaphinotus petersi biedermani*	AZ: Cochise Co., Huachuca Mts., Carr Canyon Trail / 31.4272°N, 110.3069°W elev. 2186m	048	JN639344	JN641901
*Scaphinotus petersi biedermani*	AZ: Cochise Co., Huachuca Mts., Carr Canyon Trail / 31.4272°N, 110.3069°W elev. 2186m	049	JN639345	JN641902
*Scaphinotus petersi biedermani*	AZ: Cochise Co., Huachuca Mts., Carr Canyon Trail / 31.4272°N, 110.3069°W elev. 2186m	050	JN639346	JN641903
*Scaphinotus petersi biedermani*	AZ: Cochise Co., Huachuca Mts., Carr Canyon Trail / 31.4272°N, 110.3069°W elev. 2186m	051	JN639347	JN641904
*Scaphinotus petersi biedermani*	AZ: Cochise Co., Huachuca Mts., Carr Canyon Trail / 31.4272°N, 110.3069°W elev. 2186m	052	JN639348	JN641947
*Scaphinotus petersi biedermani*	AZ: Cochise Co., Huachuca Mts., Carr Canyon Trail / 31.4272°N, 110.3069°W elev. 2186m	073	JN639367	JN641924
*Scaphinotus petersi biedermani*	AZ: Cochise Co., Huachuca Mts., Carr Canyon Trail / 31.4272°N, 110.3069°W elev. 2186m	074	JN639368	JN641925
*Scaphinotus petersi catalinae*	AZ: Pima Co., Santa Catalina Mts., Marshall Gulch / 32.4279°N, 110.7052°W elev. 2432m	042	JN639338	JN641895
*Scaphinotus petersi catalinae*	AZ: Pima Co., Santa Catalina Mts., Marshall Gulch / 32.4279°N, 110.7052°W elev. 2432m	043	JN639339	JN641896
*Scaphinotus petersi catalinae*	AZ: Pima Co., Santa Catalina Mts., Ski Valley / 32.4507°N, 110.7789°W elev. 2499m	053	JN639348	JN641905
*Scaphinotus petersi catalinae*	AZ: Pima Co., Santa Catalina Mts., Ski Valley / 32.4507°N, 110.7789°W elev. 2499m	054	JN639349	JN641906
*Scaphinotus petersi catalinae*	AZ: Pima Co., Santa Catalina Mts., Ski Valley / 32.4507°N, 110.7789°W elev. 2499m	055	JN639350	JN641907
*Scaphinotus petersi catalinae*	AZ: Pima Co., Santa Catalina Mts., Ski Valley / 32.4507°N, 110.7789°W elev. 2499m	056	JN639351	JN641908
*Scaphinotus petersi catalinae*	AZ: Pima Co., Santa Catalina Mts., Ski Valley / 32.4507°N, 110.7789°W elev. 2499m	058	JN639352	JN641909
*Scaphinotus petersi catalinae*	AZ: Pima Co., Santa Catalina Mts., Ski Valley / 32.4507°N, 110.7789°W elev. 2499m	059	JN639353	JN641910
*Scaphinotus petersi catalinae*	AZ: Pima Co., Santa Catalina Mts., Ski Valley / 32.4507°N, 110.7789°W elev. 2499m	060	JN639354	JN641911
*Scaphinotus petersi catalinae*	AZ: Pima Co., Santa Catalina Mts., Ski Valley / 32.4507°N, 110.7789°W elev. 2499m	061	JN639355	JN641912
*Scaphinotus petersi catalinae*	AZ: Pima Co., Santa Catalina Mts., Ski Valley / 32.4507°N, 110.7789°W elev. 2499m	062	JN639356	JN641913
*Scaphinotus petersi catalinae*	AZ: Pima Co., Santa Catalina Mts., Ski Valley / 32.4507°N, 110.7789°W elev. 2499m	063	JN639357	JN641914
*Scaphinotus petersi catalinae*	AZ: Pima Co., Santa Catalina Mts., Ski Valley / 32.4507°N, 110.7789°W elev. 2499m	064	JN639358	JN641915
*Scaphinotus petersi catalinae*	AZ: Pima Co., Santa Catalina Mts., Ski Valley / 32.4507°N, 110.7789°W elev. 2499m	065	JN639359	JN641916
*Scaphinotus petersi catalinae*	AZ: Pima Co., Santa Catalina Mts., Ski Valley / 32.4507°N, 110.7789°W elev. 2499m	066	JN639360	JN641917
*Scaphinotus petersi catalinae*	AZ: Pima Co., Santa Catalina Mts., Ski Valley / 32.4507°N, 110.7789°W elev. 2499m	067	JN639361	JN641918
*Scaphinotus petersi catalinae*	AZ: Pima Co., Santa Catalina Mts., Ski Valley / 32.4507°N, 110.7789°W elev. 2499m	068	JN639362	JN641919
*Scaphinotus petersi catalinae*	AZ: Pima Co., Santa Catalina Mts., Ski Valley / 32.4507°N, 110.7789°W elev. 2499m	069	JN639363	JN641920
*Scaphinotus petersi catalinae*	AZ: Pima Co., Santa Catalina Mts., Ski Valley / 32.4507°N, 110.7789°W elev. 2499m	070	JN639364	JN641921
*Scaphinotus petersi catalinae*	AZ: Pima Co., Santa Catalina Mts., Ski Valley / 32.4507°N, 110.7789°W elev. 2499m	071	JN639365	JN641922
*Scaphinotus petersi catalinae*	AZ: Pima Co., Santa Catalina Mts., Ski Valley / 32.4507°N, 110.7789°W elev. 2499m	072 /	JN639366	JN641923
*Scaphinotus petersi petersi*	AZ: Gila Co., Pinal Mts., Icehouse Canyon FTrail 198 / 33.2925°N, 110.8311°W elev. 2302.5m	081	JN639375	JN641932
*Scaphinotus petersi petersi*	AZ: Gila Co., Pinal Mts., Icehouse Canyon FTrail 198 / 33.2925°N, 110.8311°W elev. 2302.5m	082	JN639376	JN641933
*Scaphinotus petersi petersi*	AZ: Gila Co., Pinal Mts., Icehouse Canyon FTrail 198 / 33.2925°N, 110.8311°W elev. 2302.5m	083	JN639377	JN641934
*Scaphinotus petersi petersi*	AZ: Gila Co., Pinal Mts., Icehouse Canyon FTrail 198 / 33.2925°N, 110.8311°W elev. 2302.5m	084	JN639378	JN641935
*Scaphinotus petersi petersi*	AZ: Gila Co., Pinal Mts., Icehouse Canyon FTrail 198 / 33.2925°N, 110.8311°W elev. 2302.5m	085	JN639379	JN641936
*Scaphinotus petersi petersi*	AZ: Gila Co., Pinal Mts., Icehouse Canyon FTrail 198 / 33.2925°N, 110.8311°W elev. 2302.5m	086	JN639333	JN641890

### Phylogenetic reconstruction

Phylogeographic patterns were examined by inferring phylogenetic relationships from mitochondrial sequence data from all specimens collected. The combined COI and ND1 data set (2678 characters) was partitioned in five unlinked subsets (COI pos 1 and 2, COI pos 3, ND1 pos 1 and 2, ND1 pos 3, mtRNA). Maximum likelihood models were selected using MODELTEST 3.7 (Posada 2005) and likelihood searches were completed using GARLI-PART 0.97 (Zwickl 2010) using a GTR+I+G model of evolution for each subset. Other search settings were default. The searches employed a heuristic search strategy and were repeated 20 times starting from random trees keeping only the tree with the best likelihood score. Support for the relationships found in these searches was evaluated by 200 replicate bootstrap analyses with two addition sequences per replicate.

Bayesian analyses were completed in MRBAYES 3.12 (Ronquist and Huelsenbeck 2003) using four runs of 10 million generations each. The same partition strategy and model of evolution as above was used. Each run used four separate chains, sampling every 1,000 generations. Independent runs were combined using LOGCOMBINER1.5.4 (Rambaut and Drummond 2010). For each analysis, the trees in a burn-in period were excluded (the first 25% of the runs), and the majority-rule consensus tree of the remaining trees was calculated by PAUP* (Swofford 2002) to determine Bayesian Posterior Probabilities of clades. The average standard deviation of split frequencies was below 0.01 and all parameters appeared to have reached stationarity.

### Age estimates of populations

We inferred divergence dates of *Scaphinotus petersi* populations using a Bayesian relaxed clock uncorrelated lognormal method in BEAST (Drummond and Rambaut 2007) for all data combined. We partitioned the combined data into the same five subsets as used in the phylogenetic analyses. We chose unlinked GTR+I+G models with four gamma categories, a coalescent extended Bayesian skyline plot for the tree prior, and an uncorrelated lognormal relaxed clock model of rate variation estimated for each partition with a normal distribution and a mean for each gene based on the rates for each gene from Pons et al. (2010). We constrained all *Scaphinotus petersi* to be monophyletic because it was clearly monophyletic in the maximum likelihood analyses and to simplify the BEAST analyses. After an initial period of fine-tuning the operators, two separate MCMC analyses were run for 20 million generations with parameters sampled every 1000 generations. Independent runs were combined using LOGCOMBINER1.5.4 (Rambaut and Drummond 2010), and the first 30% of the generations from each run was discarded as burnin. Convergence of the chains was checked using TRACER 1.4 (Rambaut and Drummond 2007). The searches achieved adequate mixing as assessed by the high effective sampling size (ESS) values for all parameters of 100 or greater. Node ages and upper and lower bounds of the 95% highest posterior density interval for divergence times was calculated using TreeAnnotator 1.5.4 and visualized using FIGTREE 1.3.1 (Rambaut 2010).

## Results

### Phylogenetic analyses

**Figure 2.**
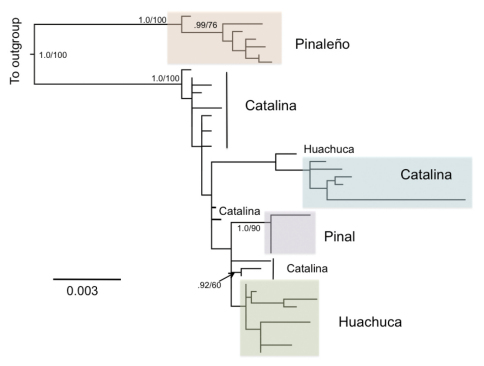
Maximum likelihood tree of *Scaphinotus petersi* populations from combined COI and ND1 data. Outgroups are removed to show greater detail. Specimen numbers are removed, but the mountain range from which they were collected is indicated. Support for branches is indicated by Bayesian Posterior Probability/Maximum Likelihood bootstrap values. Scale bar units are substitutions per site.

**Figure 3.**
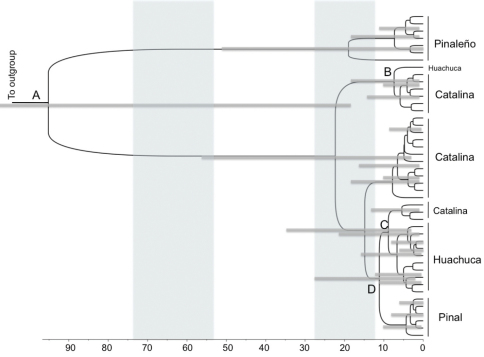
Phylogeny of *Scaphinotus petersi* dated using a Bayesian relaxed molecular clock in BEAST. Outgroups are removed to show greater detail. Specimen numbers are removed, but the mountain range from which they were collected is indicated. Branches are proportional to time in thousands of years. Shading indicates the two most recent glacial maxima. 95% confidence intervals for the ages of major clades in the tree are indicated with blue bars. The capital letters indicate population fragmentation between mountain ranges (see Table 3).

Both maximum likelihood and Bayesian analyses of mtDNA found similar topologies. The best maximum likelihood tree (Fig. 2) had a log-likelihood score of -6033.6277, and the Bayesian analysis converged on a set of trees with a mean log-likelihood score of -5797.5. Within a monophyletic *Scaphinotus petersi*, two well-supported major clades were identified, corresponding to geographic relationships between collection localities (Fig. 2) and spatially structured genetic variation at deep and shallow scales. A clade of *Scaphinotus petersi grahami* from the Pinaleño Mountains was clearly phylogenetically distinct from a western clade of *Scaphinotus petersi* from the Santa Catalina, Huachuca, and Pinal Mountains. The Santa Catalina population (*Scaphinotus petersi catalinae*) was paraphyletic with respect to a clade of *Scaphinotus petersi petersi* from the Pinal Mountains and *Scaphinotus petersi biedermani* from the Huachuca Mountains. The *Scaphinotus petersi biedermani* population did not appear to be monophyletic with one specimen grouping with members of *Scaphinotus petersi catalinae* from the Santa Catalina Mountains (Fig. 2). The overall phylogenetic tree topology estimate from GARLI and MRBAYES was similar to the BEAST analyses (Fig. 3).

**Table 2.** Primers used for DNA amplification (PCR) and sequencing for the ND1 and COI mitochodrial genes.

**Gene**	**Primer**	**Direction**	**Sequence 5’ to 3’**
Cytochrome Oxidase I (COI)	SK (modification of TY-J-1460 (Simon et al. 1994))	Forward	CGCTCTAGAACTAGTGGATCAANAAYCAYAARGAYATYG
	Pat (L2-N-3014 (Simon et al. 1994))	Reverse	TCCAATGCACTAATCTGCCATATTA
	Ron (C1-J-1751 (Simon et al. 1994))	Forward	GGATCACCTGATATAGCATTCCC
	Nancy (C1-N-2191 (Simon et al. 1994))	Reverse	CCCGGTAAAATTAAAATATAAACTTC
NADH1 dehydrogenase (ND1)	ND1F	Forward	ACATGAATTGGAGCTCGACCAGT
	16sR (LR-N-12866 (Simon et al. 1994))	Reverse	ACATGATCTGAGTTCAAACCGG

### Estimates of divergence times

Divergence time estimates for mtDNA lineages from BEAST reveal a deep and complex history of diversification (Fig. 3 and Table 3). The *Scaphinotus petersi grahami* population in the Pinaleño Mountains diverged from the western populations in this study approximately 95,200 years ago. The *Scaphinotus petersi petersi* population in the Pinal Mountains diverged from the Santa Catalina Mountain population approximately 11,000 years ago. More than one dispersal event from the Santa Catalinas to the Huachucas may have occurred about 8,900 years ago and also 7,400 years ago (Fig. 3 and Table 3).

**Table 3.** Ages of selected nodes estimated from molecular data in *Scaphinotus petersi* phylogeny from BEAST analysis. Letters correspond to nodes in Figure 3.

**Node**	**Split between populations**	**Age in years**	**95% C.I. age in years**
A	Pinaleño vs western populations	95,200	8,000–225,000
B	Huachuca vs Catalina 1	7,400	1,200–18,500
C	Huachuca vs Catalina 2	8,900	1,500–21,300
D	Catalina vs Pinal	11,200	1,800–28,200

## Discussion

### Phylogeography and genetic structure of Scaphinotus petersi

Our phylogenetic analyses indicated geographic and genetic structure within the *Scaphinotus petersi*, and most clades corresponded to isolated mountain ranges. There was strong support for two major clades in this species; an eastern clade of *Scaphinotus petersi grahami* from the Pinaleño Mountains and a western clade of *Scaphinotus petersi petersi*, *Scaphinotus petersi catalinae*, and *Scaphinotus petersi biedermani* from the Pinal Mountains, Santa Catalina Mountains, and Huachuca Mountains, respectively. While it appears the Pinaleño clade is reproductively isolated from the rest of *Scaphinotus petersi*, caution must be taken in interpreting genealogy patterns from mitochondrial data only, as it is a single locus and represents the maternal lineage only. The phylogenetic analyses suggested the Santa Catalina population is paraphyletic with respect to the Pinal and Huachuca populations that were derived from independent dispersal events from the Santa Catalinas. The history of the Huachuca population shows two relatively recent dispersal events from the Santa Catalinas to the Huachucas indicating there may have been suitable habitat in the past for low elevation Santa Catalina populations to migrate to the Huachucas. Based on morphological data, Ball (1966) suggested the Pinaleño population is fairly derived and experienced the earliest relative divergence from other *Scaphinotus petersi*, and that later, lower elevation Santa Catalina populations may have given rise to *Scaphinotus petersi petersi* and *Scaphinotus petersi biedermani* based on the pronotum and body size. Trees inferred from molecular data were in agreement with this early hypothesis.

In this study we sampled only four of the six subspecies of *Scaphinotus petersi*, and only a few of the known populations of *Scaphinotus petersi petersi*, *Scaphinotus petersi biedermani*, and *Scaphinotus petersi grahami*. Future work will include the additional subspecies and populations for a fuller picture of *Scaphinotus petersi* evolution and biogeography. We predict, with the inclusion of these samples, the phylogeography of *Scaphinotus petersi* subspecies will follow, in large part, Ball’s (1966) hypotheses of relationships based on morphological characteristics. Ball (1966) suggested the *Scaphinotus petersi grahami* from the Pinaleño Mountains shared traits with *Scaphinotus petersi kathleenae* from the Santa Rita Mountains and *Scaphinotus petersi corvus* from the Chiricahua Mountains. Thus we would predict these three subspecies form a clade even though the Santa Rita Mountains are more geographically close to the Huachuca Mountains where *Scaphinotus petersi biedermani* are found. Based on morphological similarity, Ball (1966) hypothesized *Scaphinotus petersi* in the Rincon Mountains are closely related to those in the Huachuca Mountains, however, based on the amount of dispersal from the Santa Catalina Mountains to neighboring mountain ranges and the amount of morphological variation Ball (1966) found there, we predict the population in the Rincon Mountains may be more closely related to a lineage of *Scaphinotus petersi catalinae* instead of other *Scaphinotus petersi biedermani* found in the Huachuca Mountains.

The distribution of genetic diversity in *Scaphinotus petersi* is structured across southeastern Arizona, indicating extrinsic barriers to gene flow are probably responsible for phylogeographic structure. It appears that a historical corridor of shared, linked habitat existed along a north – south ridge in the Western clade of *Scaphinotus petersi* enabling dispersal from the Santa Catalinas to the Huachuca and Pinal Mountains. This north – south ridge of connectivity pattern in biogeography has been seen in other Sky Island arthropods (Maddison and McMahon 2000, Smith and Farrell 2005a). Future phylogeographic studies will include additional populations of *Scaphinotus petersi* from Eastern and Western clades as well as closely related species in Arizona and New Mexico to further investigate the role geographic barriers have played in population isolation.

### Divergence time of isolated populations

The divergence time estimates suggested the Pinaleño population (*Scaphinotus petersi grahami*) is considerably older than the end of the last glacial period, perhaps indicating that this population was isolated during previous interglacial events in the Pliocene and persisted during Pleistocene glaciations. The western populations of *Scaphinotus petersi petersi* from the Pinals and *Scaphinotus petersi biedermani* from the Huachucas have more recent divergence times, indicating that these areas were more recently isolated, perhaps since the end of the last glacial maximum (LGM). It is important to note that the error bars for the time estimates of nodes are large, making it difficult to pinpoint with certainty divergence dates and the impact particular changes in climate have had on population isolation. Additional loci could reduce variation in estimated time to coalescence.

Ball (1966) suggested that all subspecies of *Scaphinotus petersi* could have evolved within the time span of the classical Wisconsin stage and Holocene. He hypothesized that during the pluvial stages of the Pleistocene, the montane coniferous forests occurred in the lowlands, probably along watercourses, and *Scaphinotus petersi* dispersal took place. In subsequent pluvial stages, range expansion of populations could have led to contact between previously isolated lineages. The results from our current molecular study are in concordance with this original hypothesis. During interglacial periods, contact between neighboring lineages of *Scaphinotus petersi* probably occurred in low elevation populations. These same populations were also probably the first to be extirpated during elevational shifts in habitat caused by post-glacial climate warming, leaving no signature of gene flow after the loss of these contact populations. Thus lineage boundaries like those between *Scaphinotus petersi grahami* in the Pinaleños and *Scaphinotus petersi catalinae* in the Santa Catalinas were maintained during glacial age population expansion and interglacial range retraction.

## Conclusions

Several studies have focused on the biogeography of species on the Arizona Sky Island region including plants, arthropods, birds, lizards, and mammals (Downie 2004, Linhart and Permoli 1994, McCord 1994, Sullivan 1994, Slentz et al. 1999, Barber 1999a, b, Maddison and McMahon 2000, Masta 2000, Boyd 2002, Smith and Farrell 2005a, b, McCormack et al. 2008, Tennessen and Zamudio 2008). Most of these studies have shown significant morphological variation among populations and/or genetic structure in species on the Sky Islands. However, a biogeographic study of a galling insect (Downie 2004) and a study of squirrels (Lamb et al. 1997) failed to detect evidence for genetic divergence. Past climate changes have influenced the evolution of Sky Island species in very different ways. Phylogeographic studies in other arthropods such as spiders (Masta 2000), and beetles (Smith and Farrell 2005a, b) have tested hypotheses of divergence times among isolated populations. These studies suggest ancient divergence times among populations (more than one My), suggesting a much earlier vicariance event than the proposed post-LGM habitat fragmentation. Other studies of vertebrates (Sullivan 1994, Holycross and Douglas 2007, McCormack et al. 2008) suggest a more recent post-LGM effect on population genetic structure. In addition, concordant biogeographic patterns can be seen in populations of organisms distributed on the Sky Islands. Masta (2000), Boyd (2002), and McCormack et al. (2008) all reported a North-South mountain range relationship among populations with an East-West gap.

Both recent and more ancient global climate changes could be the causal mechanisms underlying the history of habitat fragmentation in *Scaphinotus petersi*. Our results suggest *Scaphinotus petersi* populations experienced a significant fragmentation into distinct eastern and western populations separated by the San Pedro River much earlier than the last glacial period. More recently, probably after the LGM, the western populations became more fragmented in the Pinal, Santa Catalina, and Huachuca Mountains. Future work will include more populations of *Scaphinotus petersi* and closely related species from additional mountain ranges, adding missing lineages. Additional nuclear genes will be included to provide a broader picture of genetic structure and a better estimate of divergence times. These efforts will help develop a general model for understanding the phylogeographic effects of climate change in Sky Island organisms.
